# Muscle atrophy in diabetic patients with Charcot foot: a case-control study

**DOI:** 10.1007/s00256-023-04328-1

**Published:** 2023-03-30

**Authors:** Martin C. Berli, Nicolas Azaiez, Tobias Götschi, Christian W. A. Pfirrmann, Ilker Uçkay, Reto Sutter, Felix W.A. Waibel, Andrea B. Rosskopf

**Affiliations:** 1 Department of Orthopedics, Balgrist University Hospital, University of Zurich, Zurich, Switzerland; 2 Radiology, Balgrist University Hospital, University of Zurich, Forchstrasse 340, 8008 Zurich, Switzerland; 3 Unit for Clinical and Applied Research (UCAR), Balgrist University Hospital, Zurich, Switzerland; 4 Institute for Biomechanics, Swiss Federal Institute of Technology, Zurich, Switzerland; 5 MRI Medical Radiological Institute Zurich, Zurich, Switzerland

**Keywords:** Charcot foot, Muscle atrophy, Diabetes, MRI, Edema, Goutallier

## Abstract

**Purpose:**

To evaluate the distribution and severity of muscle atrophy in diabetic patients with active Charcot foot (CF) compared to diabetic patients without CF. Furthermore, to correlate the muscle atrophy with severity of CF disease.

**Material/methods:**

In this retrospective study, MR images of 35 diabetic patients (21 male, median:62.1 years ± 9.9SD) with active CF were compared with an age- and gender-matched control group of diabetic patients without CF. Two readers evaluated fatty muscle infiltration (Goutallier-classification) in the mid- and hindfoot. Furthermore, muscle trophic (cross-sectional muscle area (CSA)), intramuscular edema (none/mild versus moderate/severe), and the severity of CF disease (Balgrist Score) were assessed.

**Results:**

Interreader correlation for fatty infiltration was substantial to almost perfect (kappa-values:0.73–1.0). Frequency of fatty muscle infiltration was high in both groups (CF:97.1–100%; control:77.1–91.4%), but severe infiltration was significantly more frequent in CF patients (*p*-values: < 0.001–0.043). Muscle edema was also frequently seen in both groups, but significantly more often in the CF group (*p*-values: < 0.001–0.003). CSAs of hindfoot muscles were significantly smaller in the CF group. For the flexor digitorum brevis muscle, a cutoff value of 139 mm^2^ (sensitivity:62.9%; specificity:82.9%) in the hindfoot was found to differentiate between CF disease and the control group. No correlation was seen between fatty muscle infiltration and the Balgrist Score.

**Conclusion:**

Muscle atrophy and muscle edema are significantly more severe in diabetic patients with CF disease. Muscle atrophy does not correlate with the severity of active CF disease. A CSA < 139 mm^2^ of the flexor digitorum brevis muscle in the hindfoot may indicate CF disease.

**Supplementary Information:**

The online version contains supplementary material available at 10.1007/s00256-023-04328-1.

## Introduction

Typical complications of diabetes mellitus include micro- and macrovascular angiopathy, neuropathy, and disorders of the locomotor system, such as atrophy of skeletal muscle and loss of normal muscle function [1, 2]. A recent study showed a direct correlation between the severity of neuropathy and muscle atrophy in diabetic patients [3]. Other investigators found that muscle atrophy is indeed a feature of diabetes and is present irrespective of the presence of neuropathy in a diabetic population [2, 4–6]. Furthermore, significant muscle loss and fatty muscle infiltration in the lower limbs and the foot of diabetic patients compared to healthy controls have been stated [3, 7]. The exact pathways leading to muscle atrophy in diabetic patients have not yet been found, but in general, hyperglycemia seems to play a central role leading to an imbalance between the rate of contractile protein synthesis in the muscles and degradation [2]. Another rare complication of diabetes mellitus is the development of a so-called “Charcot foot” with a prevalence between 0.1 and 7.5% in diabetic patients [8, 9]. This inflammatory destructive neuroosteoarthropathy affects bones, joints, and soft tissues in patients with diabetes type 1 or 2 and typically results in severe foot deformities leading to a reduced quality of life [10, 11]. Muscle atrophy has also been described in cases with Charcot foot disease [12]. But, up to now, there have been no studies investigating the detailed distribution and severity of muscle changes in patients with Charcot foot disease.

The purpose of our study was to investigate muscle atrophy in diabetic patients with active Charcot foot diagnosis compared to a diabetic control group without Charcot foot. Furthermore, we looked for a potential correlation between the severity of Charcot foot disease and the severity of muscle changes.

## Materials and methods

### Study design

This retrospective case-control study was approved by the local ethics committee, the written informed consent was waived due to the retrospective study design (No. ZH-2021-02425). The MR images of all consecutive diabetic patients (*n* = 35) with a newly diagnosed active Charcot foot (included and acquired for a previously published study [13]) were used for further analysis in this study and compared to an age- and gender-matched control group. The diagnosis “newly diagnosed active Charcot foot” was established by an interdisciplinary team of orthopedic surgeons, neurologists, and radiologists in all cases. The Charcot foot was declared as “active” when swelling, redness, and hyperthermia were present. The maximum time between MR examination and Charcot diagnosis was 4 weeks. All Charcot-diagnosed patients had in-house MR images or MR images from other external institutions covering the whole foot, with at least two fluid-sensitive sequences (at least one of them with fat-saturation), and at least two T1-weighted sequences. The routine Charcot in-house protocol does not include transverse images of the forefoot. Therefore, this study focuses only on muscles in the mid- and hindfoot. The control group was selected as follows: between June 2014 and January 2022, our institutional Picture Archiving and Communication System (PACS) and patient records in our in-hospital record system (KISIM) were searched for patients with MR scans of the hindfoot and diagnosis of diabetes. Inclusion criteria for this control group were: age over 18 years, diagnosis of diabetes, external or internal acquired MR images covering the whole area from the tarsometatarsal joints to the posterior cortex of the calcaneus and including at least a T1-weighted and a fluid-sensitive sequence with fat-saturation, and age- and gender-matched to the pre-existing 35 patients with Charcot foot. Exclusion criteria for the control group were diagnosis of Charcot foot, Baxter neuropathy, immunosuppressive or osteo-anabolic medication, bone tumor or other bone destructive disease in the patient’s history, confirmed osteomyelitis or infection at time of MR examination, refusal of use of the data for research purposes, confirmed chronic muscle disease (e.g., muscular dystrophy) or spinal trauma with paraplegia or tetraplegia, paralysis of the legs due to other reasons, metal implant in the foot or history of foot surgery, and bad image quality. Finally, the first 35 patients fulfilling the age- and gender-match criteria to the Charcot group were chosen as the control group. An age match in the control group was considered fulfilled, if the age was within +/− 2 years of the case age.

### Image analysis

MR image analysis was independently performed by two readers:

Reader 1: a fellowship-trained musculoskeletal radiologist (ABR) with 14 years of experience in musculoskeletal radiology.

Reader 2: a medical student (NA), specifically trained in muscle image analysis for this analysis.

The student was trained to assess fatty muscle infiltration and to measure CSA based on 20 training cases not involved in this study. All sequences were available for the analyses. Both readers were blinded to any clinical information and each other’s reading results.

#### Muscle atrophy

**Fig. 1:**
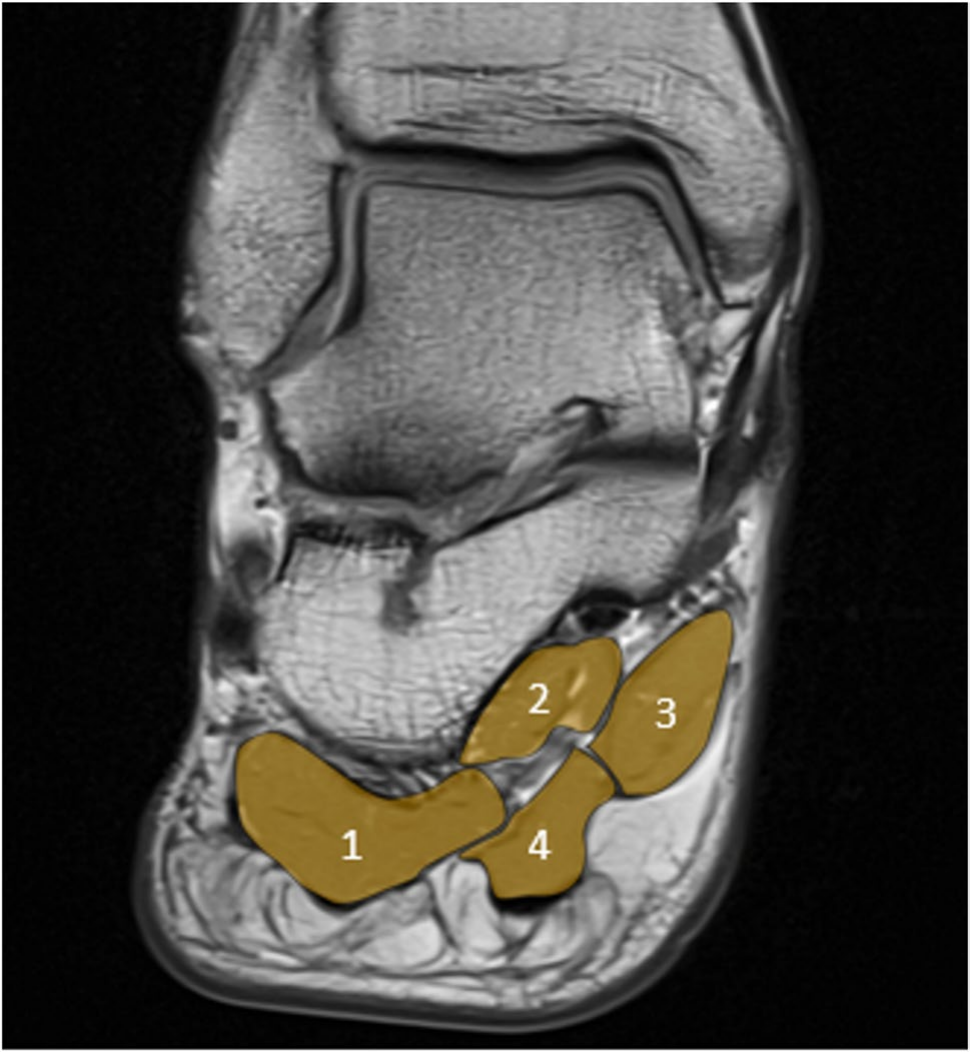
Muscles assessed in the hindfoot shown on a coronal T1-image of the hindfoot. 1= abductor digiti minimi; 2 = quadratus plantae; 3 = abductor hallucis; 4 = flexor digitorum brevis

In the hindfoot, the following muscles or muscle groups were assessed (see Fig. 1): abductor hallucis, abductor digiti minimi, quadratus plantae, and flexor digitorum brevis muscle.

**Fig. 2:**
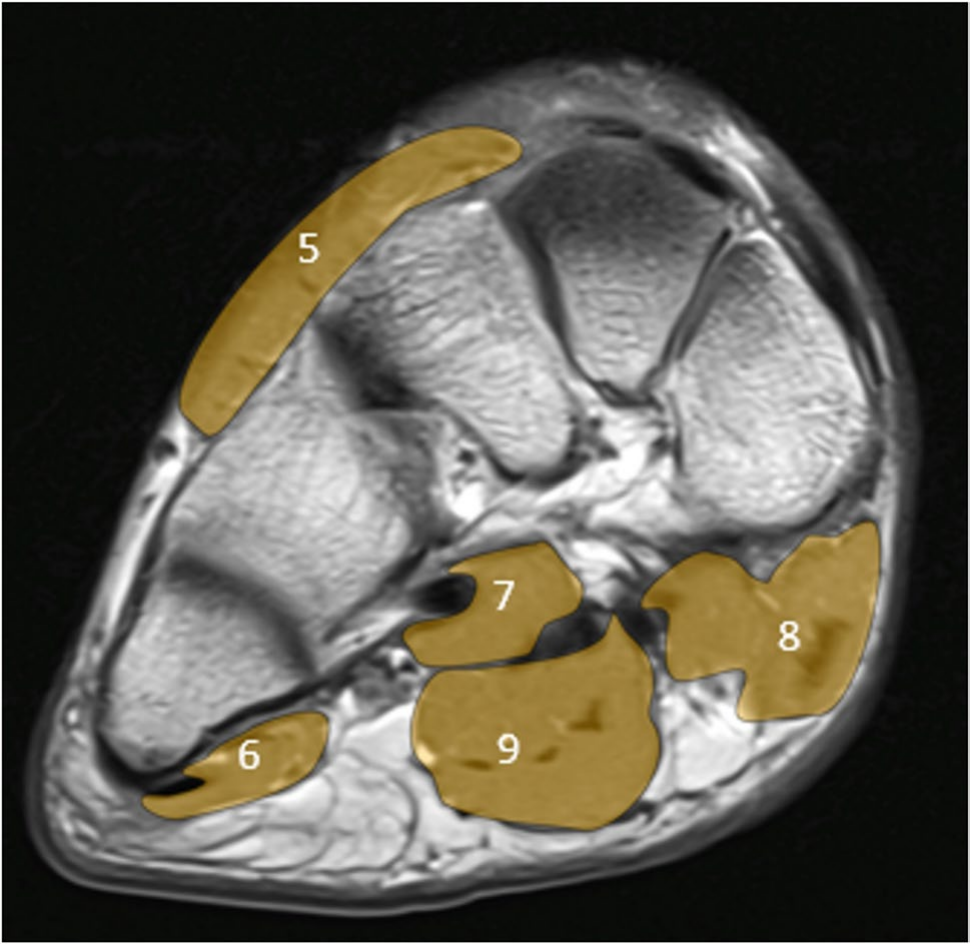
Muscles assessed in the midfoot shown on a coronal T1-image of the midfoot. 5 = extensors (extensor hallucis brevis + extensor digitorum brevis); 6 = flexor digiti minimi brevis, 7 = adductor hallucis + quadratus plantae; 8 = flexor hallucis brevis; 9 = flexor digitorum brevis

In the midfoot, the following muscles or muscle groups were assessed (see Fig. 2): flexor digitorum brevis, adductor hallucis and quadratus plantae, extensor hallucis brevis and extensor digitorum brevis, flexor digiti minimi brevis, and flexor hallucis brevis muscle.

The following parameters were assessed in all muscles:

##### Fatty infiltration

Muscle infiltration was evaluated independently by both readers.

Fatty infiltration of the muscles (regarding the average fat rate in each muscle) was assessed on T1-weighted and PD-weighted images as present or not present. In cases/controls with present fatty infiltration, the Goutallier classification [14, 15] was used for further evaluation: stage 1: Few fatty streaks within the muscle; stage 2: Less fat than muscle within the muscle; stage 3: Same amount of fat and muscle within the muscle; stage 4: More fat than muscle within the muscle. For statistical purposes, Goutallier stages 1 and 2 were summarized as “mild” infiltration, stages 3 and 4 as “severe” infiltration.

##### Cross-sectional area

**Fig. 3:**
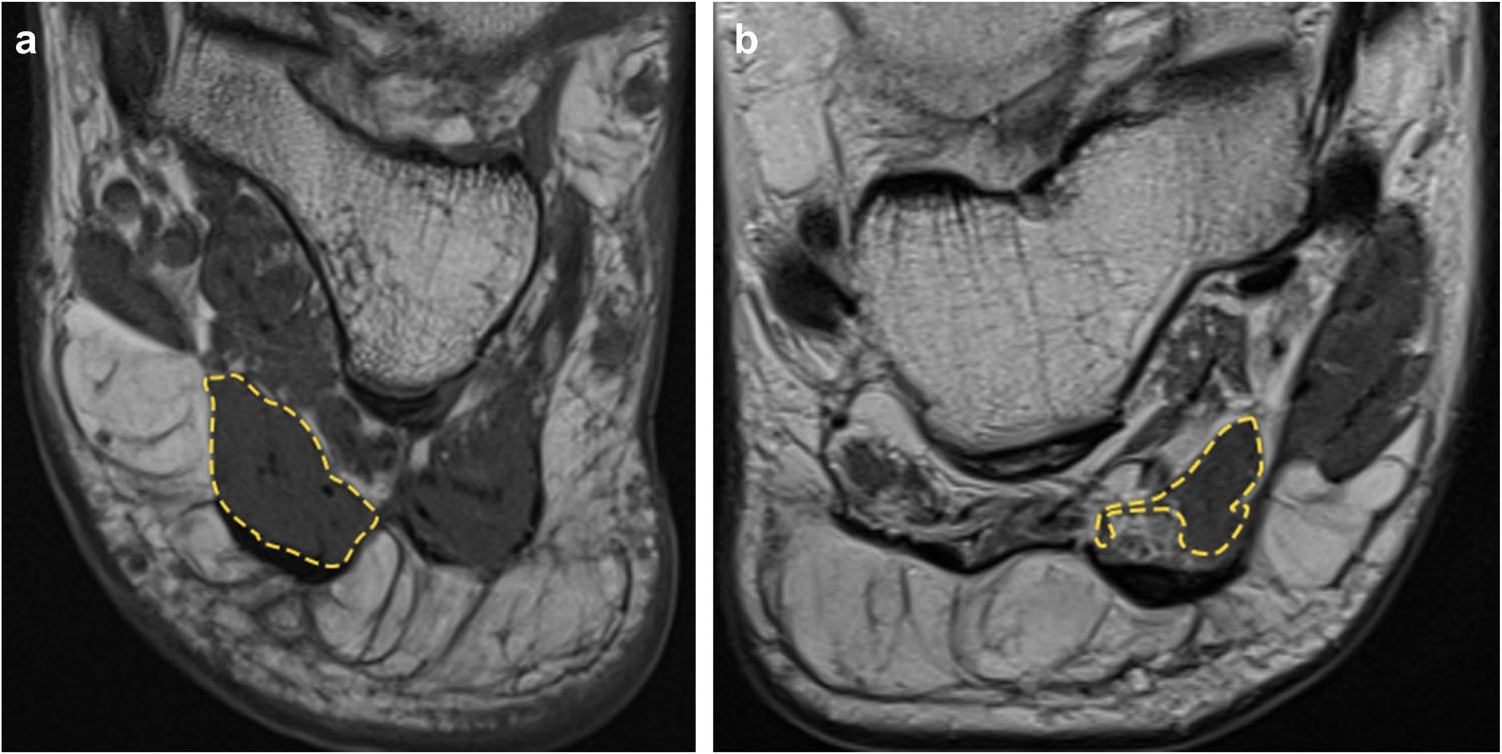
**A** CSA measurement (211.5 mm^2^) of the flexor digitorum brevis muscle in a patient with no atrophy and no fatty infiltration (Goutallier 0). **B** CSA measurement (89.7mm^2^) of the flexor digitorum brevis muscle in a patient with severe atrophy and moderate fatty infiltration (Goutallier 2). The measurements were done sparing the central tendon and including only areas with muscle tissue

Cross-sectional area (CSA) of each muscle was measured by reader 2 for evaluation of muscle atrophy (see Fig. 3). For all hindfoot muscles, the largest cross-sectional area on a coronal image (from posterior calcaneus to the navicular bone) was assessed. For all midfoot muscles the largest cross-sectional area on coronal images distally to the navicular bone was taken. We did not define specific bony or anatomic landmarks for cross-sectional assessment since the anatomy in a Charcot foot is often heavily changed due to bony collapse and joint destruction.

##### Intramuscular edema

Intramuscular signal alterations on the fluid-sensitive sequence with fat saturation were visually assessed by reader 1 using a four-point scale (0 = not present, 1 = mild, 2 = moderate, 3 = severe). In cases with heterogenous muscle signal intensity, the most severe focus of edema was scored. For statistical purposes, two major groups were formed: cases with none or mild edema versus cases with moderate or severe edema.

#### Severity of Charcot disease

Reader 1 evaluated the severity of Charcot foot cases using the Balgrist Score [13], which includes soft tissue edema, bone marrow edema, joint destruction, and fracture in regions II and III of the Sanders/Frykberg classification [16]. The maximum number of score points is 24.

### Statistical analysis

Interreader agreement was assessed using weighted Kappa coefficients with linear weights.

For group comparisons of continuous measures, Mann-Whitney *U* tests were used. Ordinal variables were compared between groups using Fisher’s exact tests. We additionally compared the presence and grading of fatty infiltration in muscles within the Charcot group using Friedmann tests. Group descriptive statistics are provided with median and interquartile ranges or in absolute and relative frequencies as applicable. Correlations were computed based on Spearman’s rank correlation tests. *p*-values below 0.05 were considered statistically significant. Muscle-specific radiological scores were additionally aggregated to yield scores for the muscle group of the hind- and the midfoot, respectively. The score of each muscle group in each case was hereby determined according to the majority of all included muscle-specific scores. Draws were resolved by assigning the higher of the two possible values. The analysis was conducted in Matlab (MATLAB, R2020a, Natick, Massachusetts: The MathWorks Inc.).

## Results

### Demographics

Since the controls were age- and gender-matched to the cases, 60% of the patients (*n* = 21) were male in both groups. The median age of the patients was 62.1 years (standard deviation:± 9.9 years; range: 44.5–84.9 years) in the Charcot group, and 63.3 years (standard deviation:± 9.7 years; range: 45.9–80.5 years) in the control group. In the Charcot group, in 54.3% of cases, the left foot was imaged versus 51,4% in the control group (*p* = 1). In the Charcot group 33 of the 35 patients (94%) had a diabetes type 2, the rest a type 1 (6%). In the control group, four patients with diabetes type 1 were found (11%) and 27 patients with type 2 (77%), and in four patients, the exact type was not stated.

### Reliability

The interreader agreement for differentiation of fatty infiltration and Goutallier staging in the mid- and hindfoot was substantial to almost perfect with kappa values between 0.734 and 1.0 (for details, see Table 1). Therefore, we chose to report only the results by reader 1 in the following section for better readability.

**Table 1:** Interreader agreement

		Kappa
Fatty infiltration Yes/no	All muscles	0.818
	Midfoot	0.746
	Hindfoot	1
Goutallier 1/2 versus 3/4	All muscles	0.734
	Midfoot	0.820
	Hindfoot	0.821

### Overall descriptives

Fatty muscle infiltration was very frequent in all assessed muscles in the control group and the Charcot group with an infiltration rate between 87.1 and 95.7% (overall: 94.1%). The most affected muscles were the abductor digiti minimi and the quadratus plantae muscle (no infiltration in only 4.3%), and the least affected muscle was the abductor hallucis (no infiltration seen in 12.9%). An advanced fatty infiltration rate (Goutallier stages 3 and 4) was seen in 23.5 to 55.0% of the evaluated muscles. A moderate/severe edema rate was seen in the evaluated muscles in 10.1–53.6%.

### Comparison between the Charcot group and the control group

#### Fatty infiltration

Fatty muscle infiltration for each muscle can be found in the Supplemental Material. Based on these measurements, percentages of fatty muscle infiltration for the hindfoot muscles, the midfoot muscles, and all muscles were calculated (Table 2). Fatty muscle infiltration was significantly higher in the Charcot group (overall muscles: *p* = 0.023). Except for one patient without fatty infiltration of the abductor hallucis muscle, fatty muscle infiltration was present in all of the mid- and hindfoot muscles in Charcot patients while the infiltration rate in the control group was between 77.1 and 91.4%. However, looking at mid- and hindfoot muscles in total, the fatty muscle infiltration was significantly higher in the midfoot muscles of Charcot muscles compared to those in the control group. In the hindfoot, no significant differences were seen (see Table 2). No significant differences were found between muscles regarding the presence of fatty infiltration within the Charcot group (*p* = 0.433).

**Table 2:** Fatty infiltration and edema

			Charcot group	Control group				Total	
			*N*	%	*N*	%	*p*-value*	*N*	%
Fatty infiltration	All muscles		35	100	29	82.9	0.023	64	91.4
	Midfoot		35	100	29	82.9	0.026	64	91.4
	Hindfoot		35	100	31	88.6	0.118	66	94.3
Goutallier	All muscles	Mild	14	40.0	26	76.5	0.004	40	58.0
		Severe	21	60.0	8	23.5		29	42.0
	Midfoot	Mild	14	40.0	25	75.8	0.003	39	57.4
		Severe	21	60.0	8	24.2		29	42.6
	Hindfoot	Mild	9	25.7	26	78.8	< 0.001	35	51.5
		Severe	26	74.3	7	21.2		33	48.5
Edema	All muscles	None/mild	22	62.9	34	97.1	0.001	56	80.0
		Moderate/severe	13	37.1	1	2.9		14	20.0
	Midfoot	None/mild	20	57.1	34	97.1	<0.001	54	77.1
		Moderate/severe	15	42.9	1	2.9		16	22.9
	Hindfoot	None/mild	22	64.7	33	94.3	0.003	55	79.7
		Moderate/Severe	12	35.3	2	5.7		14	20.3

*p-value for intergroup comparison (Charcot group versus control group)

Regarding Goutallier grading, the Charcot group showed significantly more severe fatty infiltration than the control group when comparing all assessed muscles (*p* = 0.004), muscles in the midfoot (*p* = 0.003), and muscles in the hindfoot (*p* < 0.001). Again, no inter-muscular significant differences were found within the Charcot group regarding the Goutallier grading (*p*-values: 0.095–1.0).

#### Intramuscular edema

Moderate/severe muscle edema was significantly more frequent in the Charcot group (for details, see Table 2).

Detailed evaluation for all muscles regarding fatty infiltration and muscle edema can be found in the Supplemental Material (see table S1).

#### Cross-sectional area

Median cross-sectional muscle area varied between 51.95 and 107.18 mm^2^ and was significantly smaller in all muscles in the Charcot group. The flexor digiti minimi brevis muscle showed the smallest median cross-sectional area, while the highest value was found in the abductor hallucis muscle (for details, see Table 3) in patients with Charcot foot disease.

**Table 3:** Cross-sectional area (CSA in mm^2^)

	Charcot group			Control group			
	Median	Percentile 25	Percentile 75	Median	Percentile 25	Percentile 75	*p*-value*
Flexor digitorum brevis	70.63	50.86	106.05	130.20	102.94	165.59	< 0.001
Adductor hallucis/quadratus plantae	67.24	53.32	79.21	105.62	83.45	137.59	< 0.001
Extensors	102.42	67.16	135.31	180.62	139.17	231.57	< 0.001
Flexor digiti minimi brevis	51.95	37.67	72.34	94.07	73.65	112.75	< 0.001
Flexor hallucis brevis	63.13	53.17	77.14	98.17	75.30	126.53	< 0.001
Abductor hallucis	107.18	61.49	157.45	152.86	107.41	219.07	0.002
Abductor digiti minimi	52.01	36.81	105.50	121.59	77.93	164.86	< 0.001
Quadratus plantae	58.98	47.48	90.97	92.93	73.56	114.96	< 0.001
Flexor digitorum brevis	93.35	50.87	133.68	147.37	100.01	186.89	0.001
All muscles	79.28	53.92	106.0	129.03	105.62	151.827	< 0.001
Midfoot	73.69	54.88	94.45	122.24	104.14	150.75	< 0.001
Hindfoot	76.77	49.56	116.49	142.59	102.00	154.35	< 0.001

*p-value for intergroup comparison (Charcot group versus control group)

**Fig. 4:**
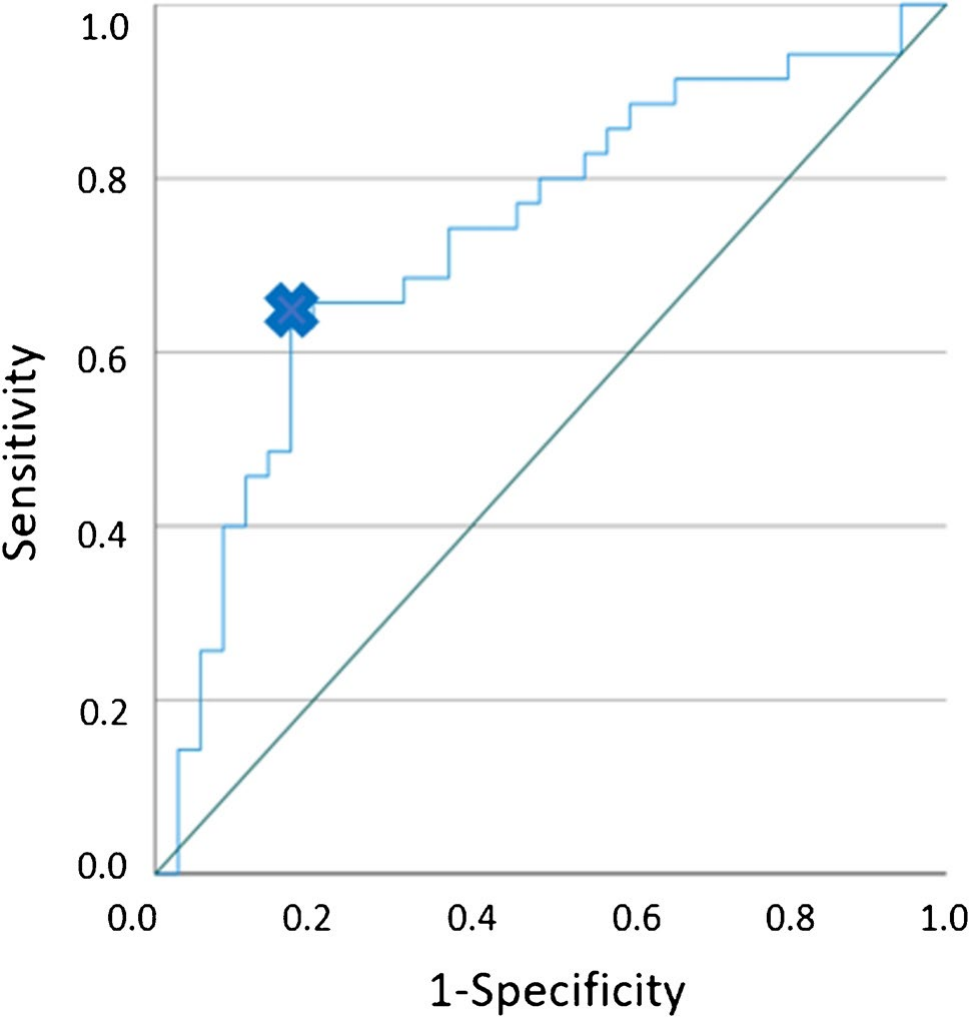
The receiver operator characteristics (ROC) for the flexor digitorum brevis muscle. The optimal threshold for prediction of a Charcot foot (blue cross) was chosen based on the distance of each point in the ROC curve to the point with 100% sensitivity and 100% specificity. The CSA cutoff value of 139 mm^2^ represents a sensitivity of 62.9% and a specificity of 82.9%

Since the hindfoot muscle borders are in general easier to detect in cases with severe atrophy, we tried to find a key muscle for differentiation between both groups: The receiver operator characteristics (ROC) curve of the flexor digitorum brevis muscle revealed an efficient cutoff value of < 139 mm^2^ for discrimination between a Charcot foot and a control (see Fig. 4). We did not choose the abductor digiti minimi muscle as key muscle since this muscle is known to frequently show even idiopathic atrophy and fatty infiltration in asymptomatic volunteers [17].

### Severity of Charcot disease

The calculated Balgrist Score values in the patients in the Charcot group were between 3 and 18 points (standard deviation: ± 3.7 points; see table S2 in Supplemental material). Spearman rank correlation showed no significant correlation between the Balgrist Score and fatty muscle infiltration (*p*-values between 0.06 and 0.49) or CSA values (*p*-values between 0.06 and 0.25). Since soft tissue edema is part of the Balgrist Score, the scores were not correlated to muscle edema in order to avoid any bias.

## Discussion

Our study results show that muscle atrophy is a frequent finding in active diabetic Charcot feet. The incidence of fatty muscle infiltration, muscle atrophy, and intramuscular edema in the hind- and midfoot is significantly higher in active diabetic Charcot feet compared to feet in patients with diabetes without Charcot disease.

It is known that patients with diabetes show an increased amount of intramuscular noncontractile tissue (e.g., muscle replaced by fatty tissue), which is highly correlated with insulin resistance and a reduction of muscle strength [18, 19]. Therefore, the high incidence of fatty muscle infiltration in both of our study subgroups is not surprising. However, the Charcot group had overall a significantly higher grade of severity of fatty muscle infiltration (Goutallier grades 3 and 4), which might be explained by a higher rate of patients with severe polyneuropathy. Unfortunately, a detailed assessment of polyneuropathy in our control group was not available. Fatty muscle infiltration was significantly increased both in the midfoot and in the hindfoot of Charcot patients compared to the control group. Unfortunately, there are no further studies available to compare the findings of fatty infiltration in patients with Charcot foot. A study by Hastings et al. [20] evaluating patients with diabetic neuropathic foot deformity (with and without medial column deformity) also found a high incidence of fatty muscle infiltration, but only in a mild to moderate severity in the assessed muscles (the intrinsic foot muscles and superficial posterior leg compartments).

In our study, the Charcot patients showed a significant loss in cross-sectional muscle area in the mid- and hindfoot. In the hindfoot, the median CSA of the control group almost doubled the size of the median CSA in the Charcot patients. This finding is consistent with another study by Andersen et al. [21] stating that the total volume of the foot muscles is halved in patients with diabetic neuropathy compared to non-neuropathic diabetic patients and healthy control subjects. Bus et al. [22] described even a 73% decrease in muscle tissue CSA in neuropathic diabetic patients compared to healthy controls. An ultrasound-based study by Severinsen et al. [23] found significantly lower CSA values of the extensor digitorum brevis muscle in patients with diabetes compared to healthy controls, and in diabetic patients with neuropathy compared to diabetics without neuropathy. The latter observation might partially explain our study results since patients with a Charcot foot usually also have a significant peripheral neuropathy.

The pathophysiologic theory for diabetes mellitus causing pronounced muscle atrophy is the unconstructive balance between the rate of contractile protein synthesis and degradation [24]. Insulin deficiency or insulin resistance of the muscle cells leads to dysregulation of protein synthesis and increased muscle degradation. Furthermore, the diabetes-related polyneuropathy results also in a neurogenic-induced muscle atrophy, pronounced in the distal extremities, e.g., the foot muscles. It is clinically important to detect atrophy of foot muscles in diabetics as the process supports thinning of sub-metatarsal fat pads and prominence of metatarsal heads, increasing the risk of foot ulcers and various foot deformities [24].

Our study findings might be of important clinical value: The diagnosis of a Charcot foot remains challenging, both for clinicians and radiologists. Especially, an early-stage Charcot foot (Eichenholtz stage 0), where frank radiographic changes are not yet present, is hard to diagnose [25–27]. Up to 95% of Charcot feet in stage 0 are missed prior to the consultation with a clinical foot specialist. However, the outcomes of stage 0 Charcot neuroarthropathy feet depend on the proper recognition and early management of the disease [25]. The therapeutical aim is to preserve the plantigrade foot axis and to prevent ulcer formation as a source of infection [28]. A delayed diagnosis leads to more frequent and more severe complications, including osteomyelitis and the need for limb amputation [29]. Nowadays, the amputation rate in tertiary care centers for patients with a Charcot foot—despite correct therapeutical support—is overall still around 7% [30]. Every additional radiologic tool may lead to an earlier diagnosis and treatment and might contribute to limb salvage. The radiologists should be alert for a possible Charcot foot in cases with diffuse, severe fatty muscle atrophy in the mid- and hindfoot of a diabetic patient with a concomitant CSA of the flexor digitorum brevis muscle < 139 mm^2^. On the other hand, a foot without or with only little fatty atrophy of the foot muscles makes the diagnosis of a possible Charcot foot very unlikely. Of course, these findings need to be proven by further prospective research studies including higher numbers of patients.

We found no correlation between the severity of Charcot foot disease (Balgrist score) and muscle atrophy and/or fatty infiltration, maybe because the changes in an active Charcot foot occur faster than the corresponding muscle changes or, simply, because severe muscle degradation is already present when the disease becomes clinically visible.

### Limitations

Our study has several limitations. Due to the retrospective study design, we could only perform qualitative analysis of fatty infiltration and no quantitative assessment in these routine MR scanning sequences was possible (no Dixon-based analysis in Charcot feet). MR examinations were limited to one point in time per patient and no follow-up images were analyzed. Furthermore, we had no data about the severity of diabetes, presence/severity of neuropathy, or other patient factors (e.g., blood glucose levels) for comparison. Moreover, there might have been a readout bias for the readers: although the readers were blinded to the patient data, the patients with Charcot foot (of course) frequently showed major foot deformities on the MR images.

About 25% of Charcot feet occur in patients without diabetes due to other reasons of peripheral neuropathy (idiopathic, alcohol abuse, vitamin deficiency, etc.). We did not investigate patients with non-diabetic Charcot feet, so our results cannot be generalized on the whole entity of Charcot arthropathy. It may also be of value for future research projects to assess muscle atrophy in other parts of the body affected by the Charcot disease, e.g., in the hands or the spine.

Future studies may also perform a longitudinal assessment of muscle atrophy in patients suffering from Charcot foot disease to elucidate the timeline regarding disease onset and progression and confirm our results for patients with non-diabetic Charcot feet.

## Conclusion

Muscle atrophy and muscle edema are significantly more severe in patients with diabetic Charcot foot disease. Muscle atrophy does not correlate with the severity of active diabetic Charcot foot disease. A CSA < 139 mm^2^ of the flexor digitorum brevis muscle in the hindfoot may indicate diabetic Charcot foot disease.

## Supplementary information

ESM 1: Supplementary Tables
