# Supplementary material for: Muscle atrophy in diabetic patients with Charcot foot: a case-control study
Source: Skeletal Radiol. 2023 Mar 30;52(9):1661–8. doi: 10.1007/s00256-023-04328-1 (PMC10348944; doi:10.1007/s00256-023-04328-1)
Supplement: Supplementary file 1 — Supplementary Tables [file 256_2023_4328_MOESM1_ESM.docx]

**Supplemental material**

Table S1

|  |  | |  | | | |
| --- | --- | --- | --- | --- | --- | --- |
|  |  |  | Charcot group | | Control group | |
|  |  |  | Count | Column N % | Count | Column N % |
| Fatty Infiltration | Flexor digitorum brevis | No | 0 | 0.0% | 5 | 14.3% |
|  |  | Yes | 35 | 100.0% | 30 | 85.7% |
|  | Adductor hallucis/quadratus plantae | No | 0 | 0.0% | 6 | 17.1% |
|  |  | Yes | 35 | 100.0% | 29 | 82.9% |
|  | Extensors | No | 0 | 0.0% | 8 | 22.9% |
|  |  | Yes | 33 | 100.0% | 27 | 77.1% |
|  | Flexor digiti minimi brevis | No | 0 | 0.0% | 4 | 11.4% |
|  |  | Yes | 35 | 100.0% | 31 | 88.6% |
|  | Flexor hallucis brevis | No | 0 | 0.0% | 5 | 14.3% |
|  |  | Yes | 35 | 100.0% | 30 | 85.7% |
|  | Abductor hallucis | No | 1 | 2.9% | 8 | 22.9% |
|  |  | Yes | 34 | 97.1% | 27 | 77.1% |
|  | Abductor digiti minimi | No | 0 | 0.0% | 3 | 8.6% |
|  |  | Yes | 35 | 100.0% | 32 | 91.4% |
|  | Quadratus plantae | No | 0 | 0.0% | 3 | 8.6% |
|  |  | Yes | 35 | 100.0% | 32 | 91.4% |
|  | Flexor digitorum brevis | No | 0 | 0.0% | 8 | 22.9% |
|  |  | Yes | 35 | 100.0% | 27 | 77.1% |
| Goutallier | Flexor digitorum brevis | Mild | 14 | 40.0% | 24 | 82.8% |
|  |  | Severe | 21 | 60.0% | 5 | 17.2% |
|  | Adductor hallucis/quadratus plantae | Mild | 16 | 45.7% | 22 | 75.9% |
|  |  | Severe | 19 | 54.3% | 7 | 24.1% |
|  | Extensors | Mild | 7 | 21.2% | 20 | 74.1% |
|  |  | Severe | 26 | 78.8% | 7 | 25.9% |
|  | Flexor digiti minimi brevis | Mild | 9 | 25.7% | 22 | 71.0% |
|  |  | Severe | 26 | 74.3% | 9 | 29.0% |
|  | Flexor hallucis brevis | Mild | 14 | 41.2% | 22 | 73.3% |
|  |  | Severe | 20 | 58.8% | 8 | 26.7% |
|  | Abductor hallucis | Mild | 16 | 47.1% | 20 | 74.1% |
|  |  | Severe | 18 | 52.9% | 7 | 25.9% |
|  | Abductor digiti minimi | Mild | 8 | 22.9% | 23 | 71.9% |
|  |  | Severe | 27 | 77.1% | 9 | 28.1% |
|  | Quadratus plantae | Mild | 9 | 25.7% | 25 | 78.1% |
|  |  | Severe | 26 | 74.3% | 7 | 21.9% |
|  | Flexor digitorum brevis | Mild | 18 | 51.4% | 22 | 81.5% |
|  |  | Severe | 17 | 48.6% | 5 | 18.5% |
| Edema | Flexor digitorum brevis | None/mild | 21 | 60.0% | 31 | 93.9% |
|  |  | Moderate/Severe | 14 | 40.0% | 2 | 6.1% |
|  | Adductor hallucis/quadratus plantae | None/mild | 15 | 42.9% | 32 | 91.4% |
|  |  | Moderate/Severe | 20 | 57.1% | 3 | 8.6% |
|  | Extensors | None/mild | 9 | 25.7% | 23 | 67.6% |
|  |  | Moderate/Severe | 26 | 74.3% | 11 | 32.4% |
|  | Flexor digiti minimi brevis | None/mild | 28 | 80.0% | 34 | 100.0% |
|  |  | Moderate/Severe | 7 | 20.0% | 0 | 0.0% |
|  | Flexor hallucis brevis | None/mild | 24 | 68.6% | 31 | 91.2% |
|  |  | Moderate/Severe | 11 | 31.4% | 3 | 8.8% |
|  | Abductor hallucis | None/mild | 25 | 73.5% | 33 | 94.3% |
|  |  | Moderate/Severe | 9 | 26.5% | 2 | 5.7% |
|  | Abductor digiti minimi | None/mild | 27 | 79.4% | 34 | 97.1% |
|  |  | Moderate/Severe | 7 | 20.6% | 1 | 2.9% |
|  | Quadratus plantae | None/mild | 19 | 55.9% | 33 | 94.3% |
|  |  | Moderate/Severe | 15 | 44.1% | 2 | 5.7% |
|  | Flexor digitorum brevis | None/mild | 24 | 70.6% | 32 | 94.1% |
|  |  | Moderate/Severe | 10 | 29.4% | 2 | 5.9% |

Note: Results Reader 1

Table S2

| Record_id | Gender | Age | Balgrist_Score |
| --- | --- | --- | --- |
| 1 | m | 60.8 | 8 |
| 2 | m | 53.0 | 7 |
| 3 | m | 74.4 | 15 |
| 4 | m | 64.3 | 7 |
| 5 | m | 68.0 | 15 |
| 6 | f | 63.8 | 12 |
| 7 | f | 70.2 | 3 |
| 8 | m | 66.7 | 14 |
| 9 | f | 68.0 | 8 |
| 10 | f | 44.5 | 5 |
| 11 | m | 67.1 | 16 |
| 12 | f | 53.1 | 7 |
| 13 | f | 58.9 | 8 |
| 14 | m | 49.6 | 10 |
| 15 | f | 79.9 | 13 |
| 16 | m | 64.5 | 7 |
| 17 | f | 60.5 | 14 |
| 18 | m | 62.8 | 6 |
| 19 | m | 51.5 | 9 |
| 20 | m | 63.3 | 4 |
| 21 | m | 63.9 | 12 |
| 22 | f | 72.0 | 11 |
| 23 | m | 48.2 | 12 |
| 24 | f | 44.6 | 12 |
| 25 | f | 51.0 | 10 |
| 26 | f | 51.8 | 7 |
| 27 | m | 48.6 | 7 |
| 28 | m | 62.1 | 10 |
| 29 | m | 52.7 | 9 |
| 30 | f | 78.4 | 4 |
| 31 | m | 84.9 | 8 |
| 32 | m | 61.3 | 18 |
| 33 | m | 64.0 | 13 |
| 34 | f | 60.1 | 8 |
| 35 | m | 57.1 | 5 |

Note: Evaluation of Balgrist Score by Reader 1.
